# Supplementary material for: Release of Melamine and Formaldehyde from Melamine-Formaldehyde Plastic Kitchenware
Source: Molecules. 2020 Aug 10;25(16):3629. doi: 10.3390/molecules25163629 (PMC7463570; doi:10.3390/molecules25163629)
Supplement: Supplementary file 1 [file molecules-25-03629-s001.pdf]

Supplementary Materials

2. Materials and Methods

2.1 Samples

The spoons used in this study are depicted in figure S1. Please note, that the display order does not correlate with the numbering in the study. We have deliberately avoided identifying the samples unambiguously, as this is only a non-representative selection of products from the market. Furthermore, the main focus of the study was on fundamental correlations, not on the actual release of the monomers.

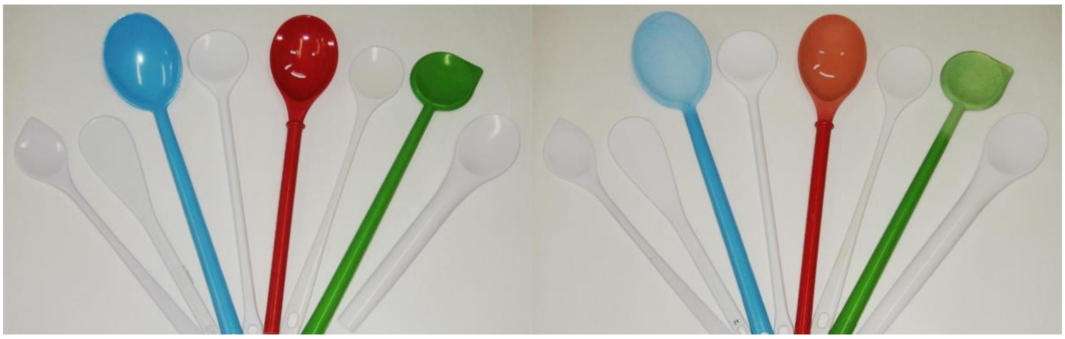

Figure S1: Spoons 1-8, left side: unused spoons, right side: spoons after cooking in 3% acetic acid.

The surface area of the spoons were determined by reducing the shapes to simple geometric figures, measurement of the dimensions with a caliper and calculation the resulting area.

Table S1: Surface area of spoons 1-8.

spoon 1	spoon 2	spoon 3	spoon 4	spoon 5	spoon 6	spoon 7	spoon 8
0.94 dm^2	0.62 dm^2	1.18 dm^2	1.16 dm^2	0.66 dm^2	0.52 dm^2	1.18 dm^2	0.79 dm^2

2.3 Migration tests

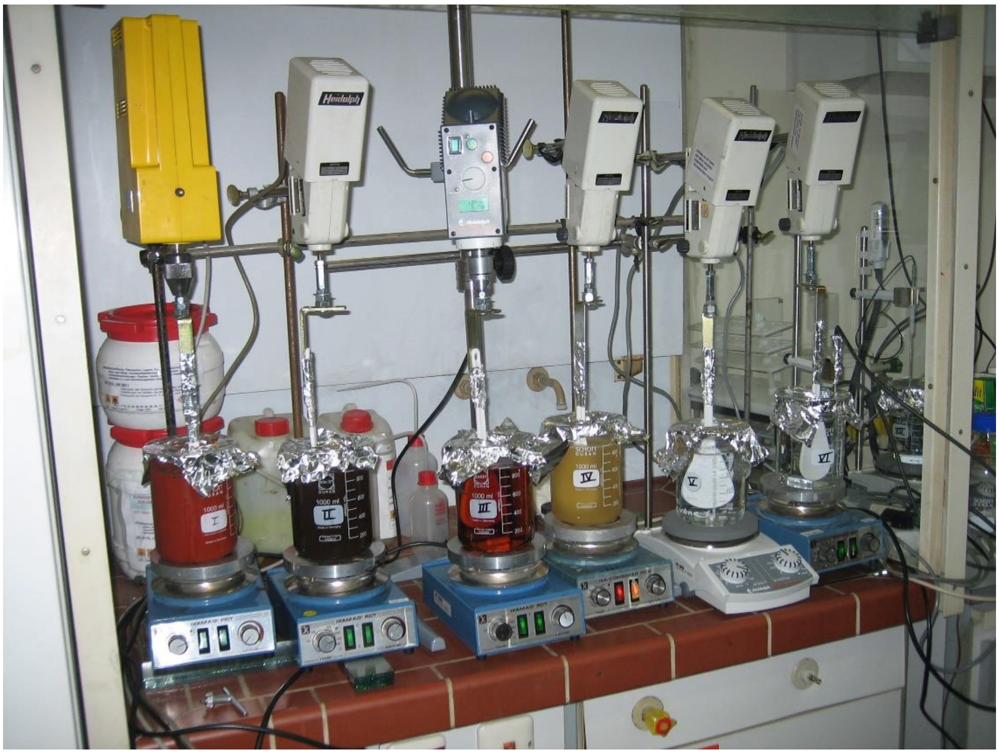

Figure S2: Stove top (hot plate) experiments of one spoon in the different matrices (from left to right: strained tomatoes, plum puree, apple juice, sauerkraut juice, 3% acetic acid and deionized water). A video of the setup can also be found in the Supplementary Materials (Video S1)

2.5 Cross Polarization/Magic Angel Spinning (CP/MAS) ^{13}C NMR

All spectra were recorded for cross-polarization times of 1 ms, 2 ms and 4 ms to ensure comparable ^{13}C CPMAS measurements results for all samples. Within a range of about 10-20% all these measurements scale equally for the samples. The three spectra are shown separately for all samples in Figure S3. A comparison of the spectra of all samples shows, that the added ^{1}H-^{13}C cross-polarization spectra are typical for the respective samples and especially that the relative signal intensities can be compared directly. Therefore, only the sum spectra of the individual samples are considered in this work.

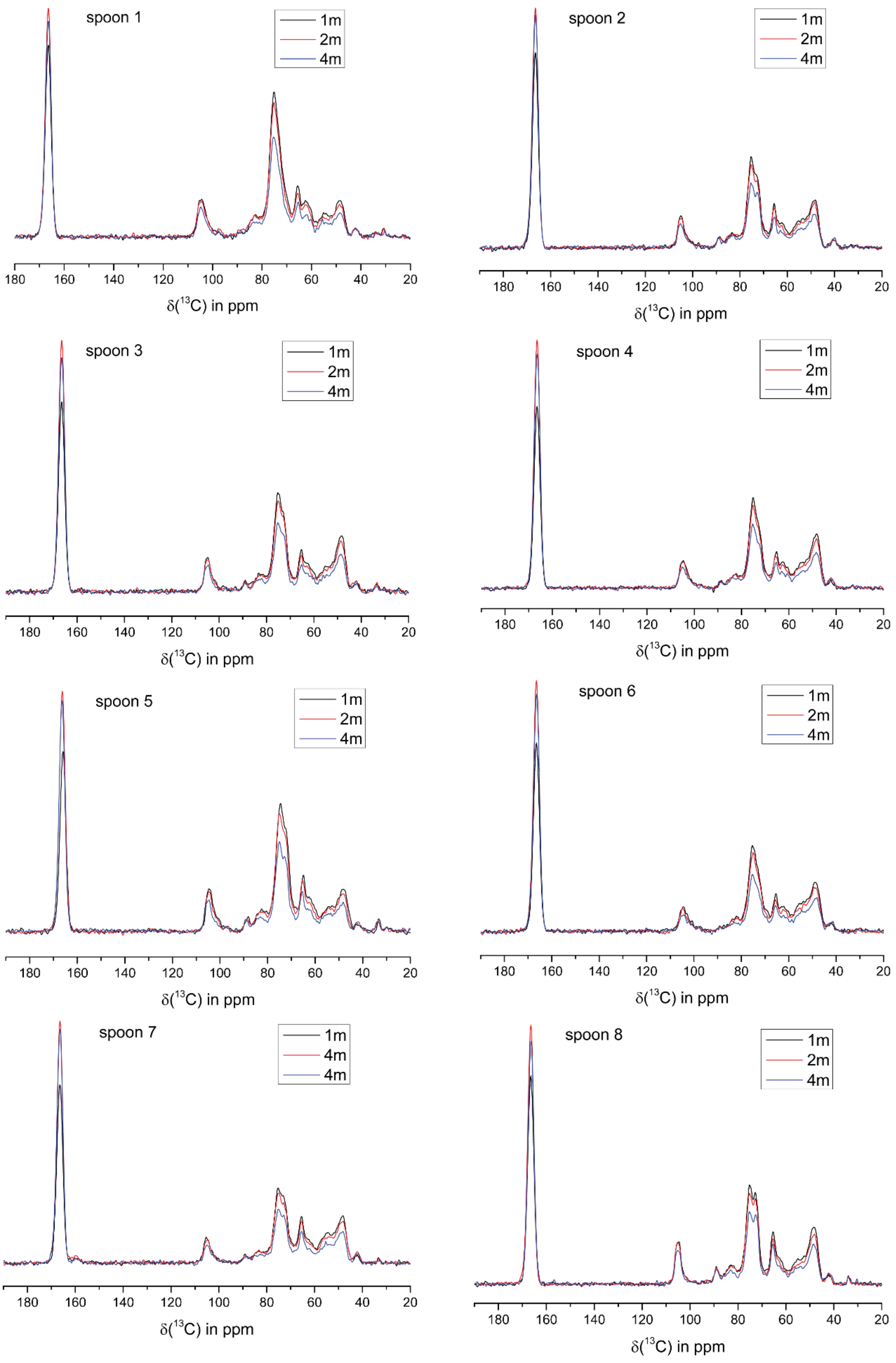

Figure S3: Comparison of the ^{13}C-CPMAS spectra for the CP times of 1 ms, 2 ms and 4 ms displayed separately for all samples.

3. Results and Discussion

3.1 Stirring of spoons during the migration

To investigate the influence of stirring the spoons a comparison to an experimental setup without stirring was done for two different sets of spoons (spoon 3 and 8). Each experiment was performed in triplicate.

Table S2: Migration of melamine and formaldehyde in mg/dm^2 from two different spoons with and without stirring the spoons (relative standard deviation in parenthesis).

	**spoon 3**		**spoon 8**	
	stirring	without stirring	stirring	without stirring
1. migration				
melamine	14.9 (10.9%)	28.1 (40.1%)	3.4 (38.4%)	9.2 (49.6%)
formaldehyde	10.6 (10.9%)	13.5 (6.5%)	7.0 (27.4%)	14.6 (35.4%)
2. migration				
melamine	38.8 (5.6%)	45.2 (-*)	26.5 (25.3%)	47.5 (27.0%)
formaldehyde	15.7 (9.9%)	17.2 (-*)	12.5 (22.7%)	20.9 (19.2%)
3. migration				
melamine	34.8 (6.5%)	39.4 (6.2%)	61.7 (27.6%)	91.3 (20.4%)
formaldehyde	14.8 (7.0%)	14.2 (12.8%)	12 (17.7%)	19.2 (15.2%)

*: only single measurement

3.2 Migration into food simulants and food

Under the conditions of the experimental set-up, it can be assumed that migrated formaldehyde partially evaporates and is no longer available for determination. To estimate the evaporation rate, formaldehyde solutions at three different concentrations (3 mg/L, 10 mg/L, 30 mg/L) covering the relevant concentration range were kept at boiling temperature for two hours and the formaldehyde concentration was monitored. The results are depicted in Figure S4.

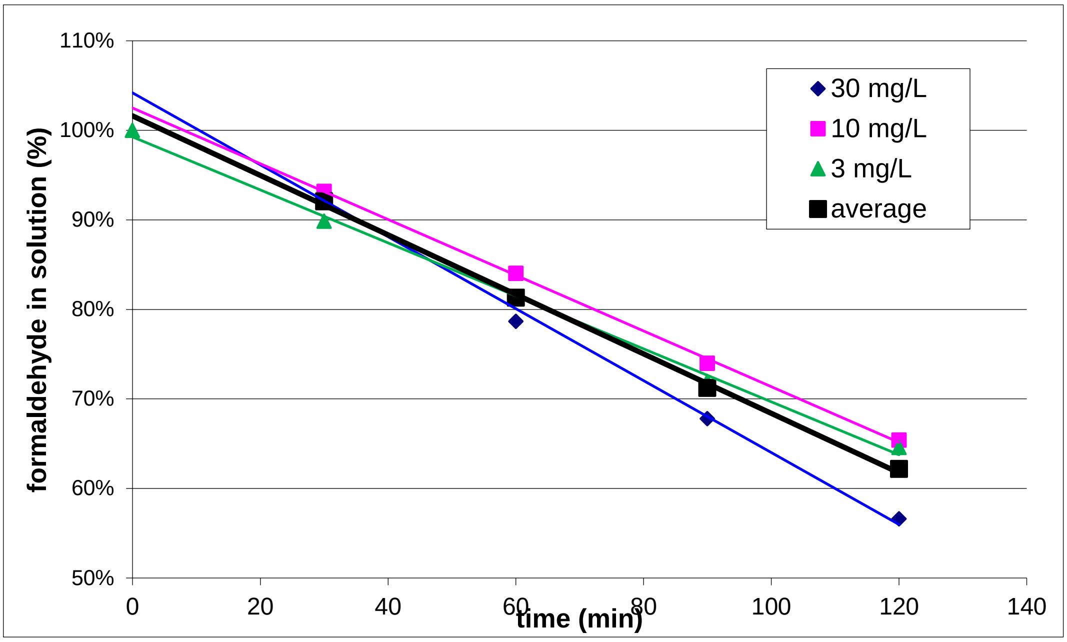

Figure S4: Relative formaldehyde content of three formaldehyde standard solutions (3 mg/L, 10 mg/L and 30 mg/L) over a boiling time of 2 h.

A linear decrease of the formaldehyde concentration is observed, giving a mean evaporation rate of 0.33% per minute. Using a spreadsheet software the cumulative evaporation was calculated using the calculated evaporation rate and one minute steps. After two hours a formaldehyde evaporation of 17.5% was calculated, resulting in a correction factor of 1.2.

3.5 NMR investigations

No correlation of the cellulose signals C1 and C2,3,5 with the melamine release in the 3rd migration is observed (Figure S5).

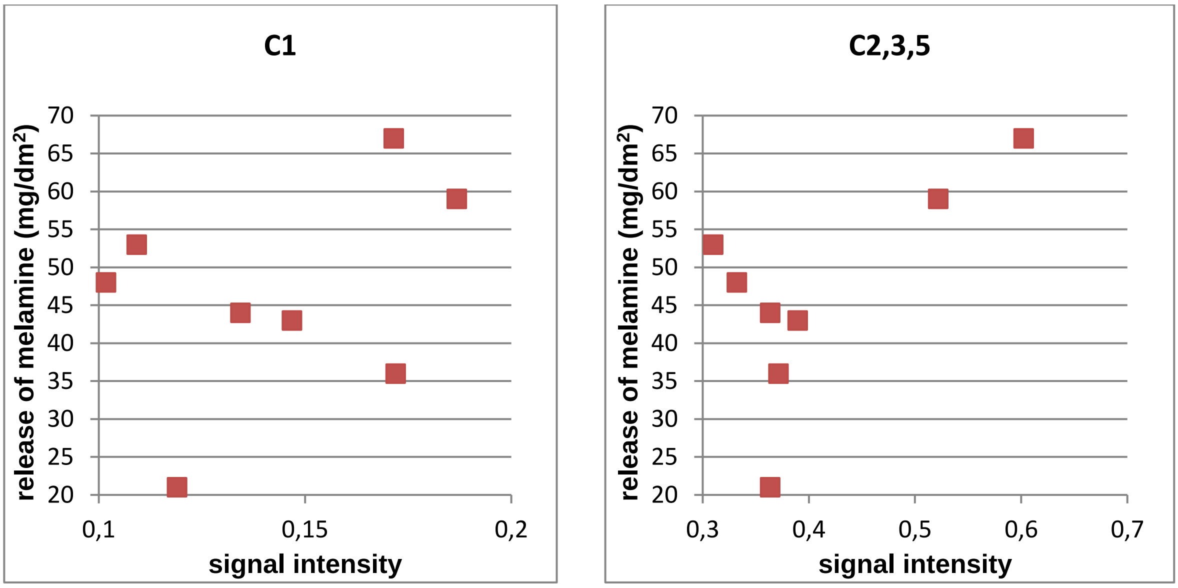

Figure S5: Correlation of the melamine release (3rd migration) on the cellulose signal intensity (left side: cellulose carbon C1 at 105 ppm, right side: cellulose carbon C2,3,5 at 70-80 ppm)

In contrast, the signal at 62-65 ppm shows a slightly positive correlation. The cellulose signal is overlaid with the signal for ether bridges. Since the results of the comparisons in Figure S5 exclude

the possibility that this correlation is due to an influence of the filler, the dependence of melamine release on the amount of ether bridges is indicated here. This confirms the results in Figure 10.

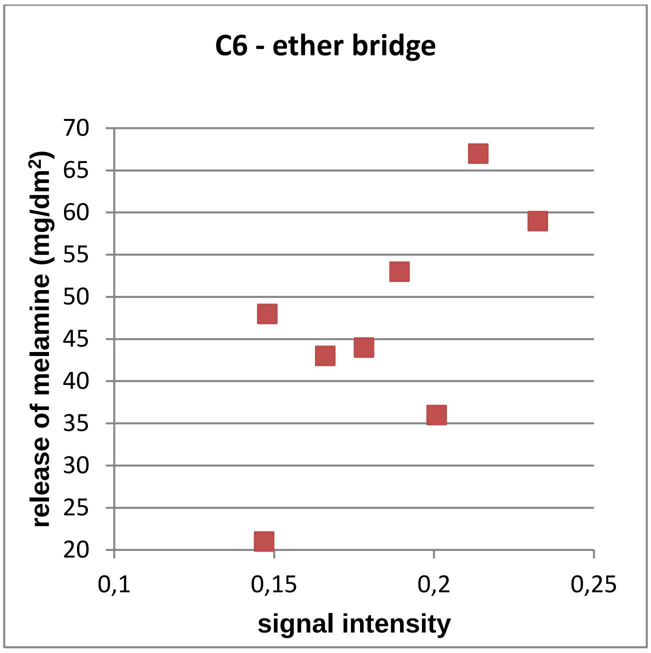

Figure S6: Correlation of the melamine release (3rd migration) on the cellulose signal intensity (cellulose carbon C6 at 62-65 ppm, overlaid with the signal for ether bridges)
